# Obesity and cardiovascular disease: Time for a paradigm shift

**DOI:** 10.1007/s12471-026-02080-4

**Published:** 2026-09-16

**Authors:** Martin E. W. Hemels

**Affiliations:** 1 Rijnstate Hospital, Arnhem, The Netherlands; 2 Radboudumc, Nijmegen, The Netherlands

The prevalence of overweight and obesity is rising globally and represents one of the greatest threats to public health. Within the Dutch healthcare system, we see the direct impact of this daily in the consulting room. Two-thirds of obesity-related excess mortality is directly attributable to cardiovascular disease (CVD) [1]. While obesity was often regarded as a secondary risk factor in the past, the current scientific evidence compels a paradigm shift: obesity is an independent, chronic disease that involves proactive and specialized cardiological care [2]. This requires a multidisciplinary approach. But how can this be embedded in daily clinical practice, and what are the priorities and main responsibilities of the disciplines involved?

For this special issue, I have invited various experts to contribute, with the aim of addressing the current scientific evidence and opportunities for optimizing state-of-the-art cardiovascular care for patients with obesity and cardiovascular disease in the Netherlands.

Lifestyle changes, including physical activity and attention to mental health, form the cornerstone of obesity treatment and are essential to reduce cardiovascular risk. *De Roos et al*. reviewed current evidence regarding the effects of exercise on cardiometabolic health and body composition in individuals with overweight and obesity, and the role of structured exercise in supporting cardiometabolic health during pharmacological treatment or surgery [3]. *Tenbült et al*. studied the effects of obesity-focused cardiac rehabilitationon on atrial fibrillation symptom burden, as part of the OPTICARE XL trial [4]. *Van Maren et al*. reviewed the effects of the addition of GLP‑1 receptor agonists on body weight and cardiovascular outcomes [5].

There is even more to read in this focused issue, and I hope it provides the readers with plenty of food for thought.
